# Angiotensin receptor type 1 and endothelin receptor type A on immune cells mediate migration and the expression of IL-8 and CCL18 when stimulated by autoantibodies from systemic sclerosis patients

**DOI:** 10.1186/ar4503

**Published:** 2014-03-11

**Authors:** Jeannine Günther, Angela Kill, Mike Oliver Becker, Harald Heidecke, Judith Rademacher, Elise Siegert, Mislav Radić, Gerd-Rüdiger Burmester, Duska Dragun, Gabriela Riemekasten

**Affiliations:** 1 Department of Rheumatology and Clinical Immunology, University Hospital Charité, Charitéplatz 1, 10117 Berlin, Germany; 2 German Rheumatism Research Center (DRFZ), Leibniz Institute, Charitéplatz 1, 10117 Berlin, Germany; 3 CellTrend GmbH, Im Biotechnologiepark, 14943 Luckenwalde, Germany; 4 Department of Rheumatology and Clinical Immunology, University Hospital Split, Soltanska 1, HR-21000 Split, Croatia; 5 Department of Nephrology, Transplantology and Intensive Care, University Hospital Charité, Augustenburger Platz 1, 13353 Berlin, Germany

## Abstract

**Introduction:**

Agonistic autoantibodies (Aabs) against the angiotensin II receptor type 1 (AT1R) and the endothelin receptor type A (ETAR) have been identified in patients with systemic sclerosis (SSc). In our present study, we examined the expression of the AT1R and the ETAR in human immune cells and the pathological effects mediated through these receptors by their corresponding Aabs.

**Methods:**

Protein expression of AT1R and ETAR on peripheral blood mononuclear cells (PBMCs) from healthy individuals and SSc patients was analyzed using flow cytometry, and mRNA expression of both receptors in PBMCs from healthy donors was examined by real-time PCR. In addition, PBMCs from healthy donors were stimulated *in vitro* with affinity-purified immunoglobulin G (IgG) fractions from SSc patients positive for AT1R and ETAR Aabs, as well as with IgG from healthy donors serving as controls. Alterations in cell surface marker expression, cytokine secretion and chemotactic motility were analyzed using flow cytometry, enzyme-linked immunosorbent assays and chemotaxis assays, respectively. The results were correlated with the characteristics and clinical findings of the IgG donors.

**Results:**

Both AT1R and ETAR were expressed on PBMCs in humans. Protein expression of both receptors was decreased in SSc patients compared with that of healthy donors and declined during the course of disease. IgG fractions of SSc patients positive for AT1R and ETAR Aabs induced T-cell migration in an Aab level–dependent manner. Moreover, IgG of SSc patients stimulated PBMCs to produce more interleukin 8 (IL-8) and chemokine (C-C motif) ligand 18 (CCL18) than did the IgG of healthy donors. All effects were significantly reduced by selective AT1R and ETAR antagonists. Statistical analysis revealed an association of SSc-IgG induced high IL-8 concentrations with an early disease stage and of high CCL18 concentrations with lung fibrosis onset and vascular complications in the respective IgG donors.

**Conclusion:**

In our present study, we could demonstrate the expression of both AT1R and ETAR on human peripheral T cells, B cells and monocytes. The decreased receptor expression in SSc patients, the inflammatory and profibrotic effects upon Aab stimulation of PBMCs *in vitro* and the associations with clinical findings suggest a role for Aab-induced activation of immune cells mediated by the AT1R and the ETAR in the pathogenesis or even the onset of the disease.

## Introduction

Systemic sclerosis (SSc) is a severe rheumatic disease characterized by widespread fibrosis, vasculopathy and high serum levels of agonistic autoantibodies (Aabs) [1,2]. Activation of the immune system has a crucial role in SSc pathophysiology [3,4], and perivascular mononuclear cell infiltrates are found before any histological evidence of fibrosis [5]. These infiltrates are usually associated with an early disease stage, and factors that are involved in the progressive vasculopathy remain poorly defined [6]. Agonistic Aabs against the angiotensin II receptor type 1 (AT1R) and the endothelin receptor type A (ETAR) were recently identified in the sera of SSc patients and are considered to contribute to the pathogenesis of the disease [2]. As is typical of G protein–coupled receptors (GPCRs), activation of AT1R and ETAR initiates several physiological and pathophysiological processes [7-9]. Angiotensin II (Ang II), the major biologically active peptide of the renin-angiotensin system, mediates its effect through the AT1R and the angiotensin receptor type 2 (AT2R). Besides its hemodynamic properties, Ang II can contribute to inflammation and fibrosis [7-9], primarily mediated through the AT1R [10,11]. Endothelin is a strong vasoconstrictor. Its known receptors are the ETAR and the endothelin receptor type B. The isoform endothelin 1 (ET-1), the most abundant endothelin isoform in human plasma, shows the highest affinity for the ETAR and is a potent mediator of inflammation in the vasculature [12,13].

Inflammation belongs to the first events in the pathogenesis of SSc, leading to vascular damage and fibrosis. Several growth factors and cytokines involved in these processes are altered in the serum and affected tissue of SSc patients. Serum levels of Ang II and ET-1 are elevated and have been shown to promote vascular injury and fibrosis in SSc patients [14,15]. Thus, Ang II and ET-1 appear to contribute to the pathogenesis of SSc. Therefore, it is likely that Aabs, which act agonistically through the AT1R and the ETAR, could drive the pathophysiological process in SSc.

The expression of the AT1R in human peripheral blood mononuclear cells (PBMCs) has been described previously [8,16], but its function in immune cells remains poorly understood. It has been demonstrated that activation of the AT1R leads to a reduction in the activation threshold of murine T cells [17] and to a higher chemotactic motility of human T cells, dendritic cells and natural killer cells [8,18].

In our present study, we hypothesized that Aabs against AT1R and ETAR could activate immune cells expressing these receptors and thereby provide a link between the adaptive and innate immune system. We analyzed the differential expression of both receptors on human T cells, B cells and monocytes from peripheral blood of healthy donors and SSc patients. In addition, we investigated the effects of the Aabs against these receptors on human peripheral immune cells, the associations of the experimental results with clinical findings and hence the possible role of anti-AT1R and anti-ETAR Aabs in the pathogenesis of SSc.

## Methods

### Antibodies and reagents

Rabbit polyclonal immunoglobulin G (IgG) anti-human AT1R (N-10: sc-1173), rabbit polyclonal IgG anti-human ETAR (H-60: sc-33535) and rabbit polyclonal IgG isotype control (sc-3888) were obtained from Santa Cruz Biotechnology (Santa Cruz, CA, USA). Goat anti-rabbit Alexa Fluor 430 (A-11064) was obtained from Invitrogen (Carlsbad, CA, USA) as a secondary antibody. Mouse IgG anti-human CD3 phycoerythrin (1273; clone UCHT-1), mouse IgG anti-human CD19 cyanine 5 (Cy5) (1624 clone BU12), mouse IgG anti-human CD14 fluorescein isothiocyanate (FITC) (1353 clone TM1) and mouse IgG anti-human CD14 Cy5 (1744 clone TM1) were obtained from the German Rheumatism Research Center (DRFZ; Berlin). Mouse IgG anti-human CD16 FITC (555406) was obtained from BD Biosciences (Heidelberg, Germany). Chemicals and reagents were purchased from Sigma-Aldrich (Munich, Germany) unless stated otherwise.

### Patients

Eighteen patients (mean age ± SD = 48.4 ± 11.3 years, 10 females and 8 males, 17 diffuse SSc and 1 limited SSc) and fourteen healthy donors (mean age ± SD = 41.1 ± 15.3 years, 8 females and 6 males) were enrolled in our study of AT1R and ETAR expression in PBMCs. IgG was isolated from 32 patients (mean age = 57.9 ± 13.5 years, 24 females and 9 males, 19 diffuse SSc and 14 limited SSc) and 10 healthy donors (mean age = 46.5 ± 6.3 years, 10 females and 0 males). All patients had an established diagnosis of SSc according to the SSc criteria of the American College of Rheumatology [19]. The study was conducted in accordance with the Declaration of Helsinki and was approved by the ethical committee of the University Hospital Charité, Berlin. The patients gave us their written informed consent to participate (EA1/042/09, EA1/013/05 and EA1/160/10).

The clinical subsets of diffuse SSc and limited SSc were defined according to the classification system described previously [20]. Clinical assessment was performed using protocols standardized by the European Scleroderma Trials and Research group [21].

### Isolation of peripheral blood mononuclear cells and immunoglobulin G

For PBMC isolation, venous blood was collected into heparinized tubes (BD Vacutainer Systems, Plymouth, UK). PBMCs were prepared by centrifugation through a density gradient on LSM1077 Lymphocyte Separation Medium (PAA Laboratories, Pasching, Austria). To prepare cells for RNA extraction, monocytes and lymphocytes were isolated from the PBMCs of healthy donors by magnetic-activated cell sorting according to the manufacturer’s instructions. Monocytes were isolated by depletion of nonmonocytes with the Monocyte Isolation Kit II, and T cells and B cells were positively selected by means of anti-CD3 and anti-CD19 magnetic beads, respectively (all obtained from Miltenyi Biotec, Bergisch Gladbach, Germany). For chemotaxis assays, T cells were isolated by depletion of non–T cells by performing magnetic-activated cell sorting with the Pan T Cell Kit (Miltenyi Biotec). The purity of the selected populations was usually higher than 95% as assessed by flow cytometry.

For IgG isolation, venous blood was collected into ethylenediaminetetraacetic acid tubes (BD Vacutainer Systems) and centrifuged to harvest the serum. IgG was isolated by protein G sepharose chromatography in 20 mM phosphate buffer, pH 7.0. IgG was eluted with 0.1 M glycine HCl, pH 2.7, and pH was neutralized with 1 M Tris-HCl, pH 9.0. Eluted IgG was dialyzed against phosphate-buffered saline (PBS). To determine protein concentration, absorbance was measured at 280 nm (EMax Endpoint ELISA Microplate Reader; Molecular Devices, Sunnyvale, CA, USA). Anti-AT1R/ETAR titers were detected in purified IgG as well as in serum by solid-phase assay in cooperation with CellTrend GmbH (Luckenwalde, Germany) using a commercially available enzyme-linked immunosorbent assay (ELISA) kit (One Lambda, Canoga Park, CA, USA) as previously described [2].

### Flow cytometry

PBMCs were incubated in 1:10 diluted Flebogamma (Instituto Grifols, Barcelona, Spain) to block nonspecific binding sites. Afterward cells were incubated with the appropriate concentrations of surface marker antibodies in a PBS/bovine serum albumin (BSA) solution for 20 minutes at 4°C. Prior to intracellular staining, cells were washed with PBS, fixed with 4% formaldehyde and permeabilized with 0.5% saponin. For receptor staining, cells were incubated with the primary antibodies or the isotype control, washed with PBS/BSA and incubated with the secondary antibody, both for 45 minutes on ice. Cytometric measurements were carried out using a LSR II flow cytometer (BD Biosciences) and analyzed with FCS Express software (DeNovo Software, Los Angeles, CA, USA) and FlowJo software (TreeStar, Ashland, OR, USA).

### RNA, cDNA and real-time PCR

mRNA was isolated by NucleoSpin RNA II (Macherey-Nagel, Düren, Germany) and cDNA was generated by Moloney murine leukemia virus reverse transcriptase (Promega, Mannheim, Germany), each according to the manufacturer’s instructions. Real-time PCR reagents contained 5 μl of cDNA, 0.25 mM deoxynucleotide triphosphates (Bioline, Luckenwalde, Germany), 12 μg/ml BSA, 1× SYBR Green I (Molecular Probes, Darmstadt, Germany), 1 U of Immolase DNA Polymerase (Bioline), 500 mM Tris, pH 8.8, 4 and 6 mM MgCl_2_, respectively, and 0.5 nmol/ml primer mix (TIB MOLBIOL, Berlin, Germany). Reactions were performed in an Mx3000P quantitative PCR thermal cycler (Stratagene, Waldbronn, Germany). Primers were designed using Primer Express 2.0 software (Applied Biosystems): AT1R (*AGTR1*) forward: 5′-TTC AGC CAG CGT CAG TTT CA-3′, reverse: 5′-GGC-GGG-ACT-TCA-TTG-GGT-3′; ETAR (*EDNRA*) forward: 5′-CAC AGA GCT CAG CTT CCT GGT TA-3′, reverse: 5′-GTA ATT TTA GTC TGC TGT GGG CAA TAG-3′; and eukaryotic elongation factor 1 α1 (EF1A; housekeeping gene *EEF1A1*) forward: 5′-GTT GAT ATG GTT CCT GGC AAG C-3′, reverse: 5′-GCC AGC TCC AGC AGC CTT C-3′.

### T-cell culture and chemotaxis assays

Isolated T cells were cultured overnight at a concentration of 1 to 2 million cells/ml in RPMI 1640 medium with GlutaMAX (Gibco/Life Technologies, Grand Island, NY, USA), 1% IgG-free fetal calf serum (FCS), 100 U/ml penicillin and 100 µg/ml streptomycin (GE Healthcare Life Sciences, Vienna, Austria) in a humidified atmosphere with 5% CO_2_ at 37°C. On the next day, cells were seeded at a concentration of 1.0 to 1.3 million cells/ml in anti-CD3/anti-CD28–coated well plates (10 and 5 μg/ml, respectively, coated overnight at 4°C) and cultured for 3 days in the presence of 10 μg/ml interleukin 2 (IL-2) under the conditions described above. Cells were washed in PBS/BSA and resuspended in assay medium (RPMI 1640 with 0.5% BSA). Chemotaxis experiments were performed in transwell cell culture chambers with a polycarbonate membrane of 5-μm pore size (Costar; Corning Life Sciences, Corning, NY, USA). A total of 600,000 cells in 100 μl of the assay medium was placed into the inserts. The inserts were positioned into individual wells containing 600 μl of assay medium with or without 500 μg/ml IgG as indicated. The AT1R antagonist valsartan (provided by Dominik N Müller, Max-Delbrück-Centre for Molecular Medicine, Berlin, Germany) or the ETAR antagonist sitaxsentan (provided by Pfizer, New York, NY, USA) was added as indicated into both the inserts and the corresponding wells. Valsartan and sitaxsentan were used at concentrations of 10^-5^ M and 10^-4^ M, respectively. Appropriate concentrations of the antagonists and IgG were determined in test experiments using Ang II and ET-1. Plates were incubated for 2 hours in a humidified atmosphere with 5% CO_2_ at 37°C. The number of T cells that had migrated into the well was quantified with a Neubauer hemocytometer (Marienfeld Superior, Lauda-Königshofen, Germany).

Migration of PBMCs was examined with freshly isolated cells in transwell cell culture chambers with a collagen-coated polycarbonate membrane of 3-μm pore size (Costar; Corning Life Sciences). A total of 200,000 cells in 50 μl was placed into the inserts, and assays were performed as described above. Migrated cells were quantified using the adenosintriphosphate monitoring ATPlite Luminescence Assay System (PerkinElmer, Waltham, MA, USA) according to the manufacturer’s instructions.

### Cell culture and stimulation of peripheral blood mononuclear cells

Isolated PBMCs were cultured at a concentration of 1 to 2 million cells/ml in RPMI 1640 medium with GlutaMAX, 1% IgG-free FCS, 100 U/ml penicillin and 100 μg/ml streptomycin (GE Healthcare Life Sciences) in a humidified atmosphere with 5% CO_2_ at 37°C. Valsartan or sitaxsentan was added to the cells at a concentration of 10^-5^ and 6 × 10^-5^, respectively. After incubation in 1:10 diluted Flebogamma (Instituto Grifols) to block nonspecific binding sites, cells were treated with 1 mg/ml IgG as indicated. Cell viability and appropriate concentrations of the antagonists and IgG were determined in test experiments. Cells and supernatants were collected after 8 and 20 hours, respectively, depending on the outcome parameter, and then subjected to fluorescence-activated cell-sorting analysis, real-time PCR or ELISA. To detach adherent monocytes, they were washed with PBS and incubated with Accutase (Innovative Cell Technologies, San Diego, CA, USA) for 5 to 15 minutes at 37°C.

### ELISA

Anti-AT1R and anti-ETAR Aabs were detected by using a commercially available sandwich ELISA (One Lambda) as described previously [2]. Because serum levels can differ from those of purified IgG, we tested Aab levels in IgG fractions after purification. Anti-AT1R and anti-ETAR Aab levels of IgG fractions from healthy donors were usually below 10 arbitrary units, whereas those from SSc patients ranged up to 40 arbitrary units.

Supernatants of treated PBMCs were collected after 8 and 20 hours, respectively, depending on the outcome parameter. Cytokines and soluble proteins were analyzed by performing sandwich ELISAs in a colorimetric detection system with tetramethylbenzidine, hydrogen peroxide and horseradish peroxidase. For the detection of IL-8, renamed chemokine (CXC motif) ligand 8 (CXCL8), the anti-human IL-8 capture antibody H8A5 (Biolegend) and the biotin-coupled detection antibody E8N1 (Biolegend) were used according to the manufacturer’s instructions. To analyze the concentrations of chemokine (C-C motif) ligand 18 (CCL18), transforming growth factor β1 (TGF-β1), soluble CD62 (sCD62) and soluble CD14 (sCD14), duoset kits were used according to the manufacturer’s instructions (R&D Systems, Minneapolis, MN, USA). Absorbance was measured in a microplate reader at 450 nm and analyzed using SoftMax Pro version 5 software (Molecular Devices).

### Statistical analysis

Data were evaluated statistically using GraphPad Prism 5 software (GraphPad Software, La Jolla, CA, USA). Statistical significance was determined by the Mann-Whitney *U* test, the Wilcoxon matched-pairs test and Spearman’s correlation analysis. A value of *P* < 0.05 was interpreted as statistically significant.

## Results

### AT1R and ETAR are expressed in human PBMCs, and their protein expression is decreased in SSc patients

**Figure 1:**
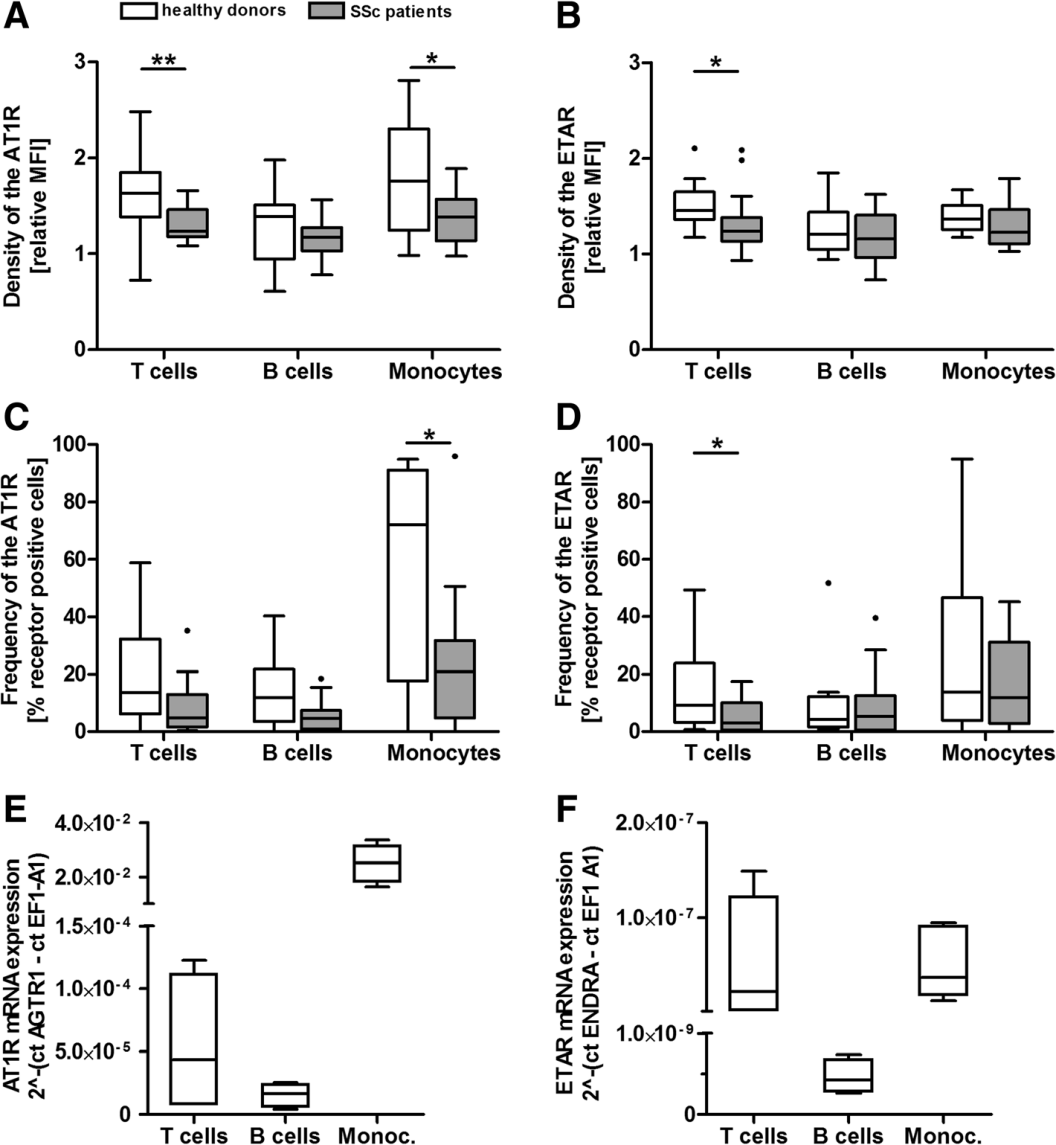
**Angiotensin II receptor type 1 and endothelin receptor type A are expressed in human peripheral blood mononuclear cells, their protein expression is decreased in systemic sclerosis patients.** Protein expression of both receptors in CD3+ T cells, CD19+ B cells and CD14+ monocytes of systemic sclerosis (SSc) patients (*n* = 18) and healthy donors (*n* = 14) was measured by flow cytometry. **(A)** Density of angiotensin II receptor type 1 (AT1R) and endothelin receptor type A (ETAR) **(B)** is represented by the median fluorescence intensity (MFI) normalized to the isotype control. **(C)** Frequency of AT1R-positive cells and **(D)**  ETAR-positive cells is represented by the percentage relative to an isotype control. **(E)** AT1R mRNA (*AGTR1*) expression and **(F)** ETAR mRNA (*ENDRA*) expression in CD3+ T cells, CD19+ B cells and CD14+ monocytes of healthy donors (*n* = 3) was measured by real-time PCR and calculated using the 2^-ΔCt^ method. Results are represented relative to the housekeeping gene elongation factor 1 α1 (*EF1A1*). Statistical analysis was carried out by Mann–Whitney *U* test. Data are shown as box-and-whisker plots (Tukey). *P < 0.05 and **P < 0.01.

Protein expression of AT1R and ETAR was measured on T cells, B cells and monocytes by flow cytometry (Figures 1A to 1D). In healthy donors, the density of both receptors (represented by open boxes Figures 1A and 1B) varied from a median fluorescence intensity (MFI) of 1.3 to 1.8 relative to the isotype control in all analyzed subsets and was lowest on B cells. The frequency of AT1R-positive cells showed median values of 14% for T cells, 12% for B cells and 73% for monocytes (represented by open boxes in Figure 1C). The frequency of ETAR-positive cells showed median values of 10% for T cells, 5% for B cells and 17% for monocytes (open boxes Figure 1D). The frequency range of AT1R- and ETAR-positive cells was large, especially in monocytes. Protein expression data of healthy donors were confirmed by real-time PCR to analyze mRNA expression of the receptors in isolated T cells, B cells and monocytes (Figures 1E and 1F).

Chronic receptor activation often results in downregulation. Therefore, the expression of the receptors was studied on PBMCs of SSc patients in whom chronic activation could be assumed because of the presence of anti-AT1R/anti-ETAR Aabs in these patients. As shown in Figure 1A, AT1R density was significantly decreased on CD3+ T cells and CD14+ monocytes of SSc patients (*P* < 0.01 and *P* < 0.05, respectively) compared with those of healthy donors. In addition, the frequency of AT1R-positive CD14+ monocytes (Figure 1C) was significantly decreased (*P* < 0.05). The ETAR density and frequency of ETAR-positive cells (Figures 1B and 1D) were significantly decreased on the CD3+ T cells of SSc patients compared with those of healthy donors (*P* < 0.05).

Because of the high number of cells necessary to analyze mRNA expression of both receptors in different PBMC subsets, we did not perform real-time PCR with PBMCs of SSc patients.

### Protein expression of AT1R and ETAR correlates negatively with disease duration in SSc patients and age in healthy individuals

**Figure 2:**
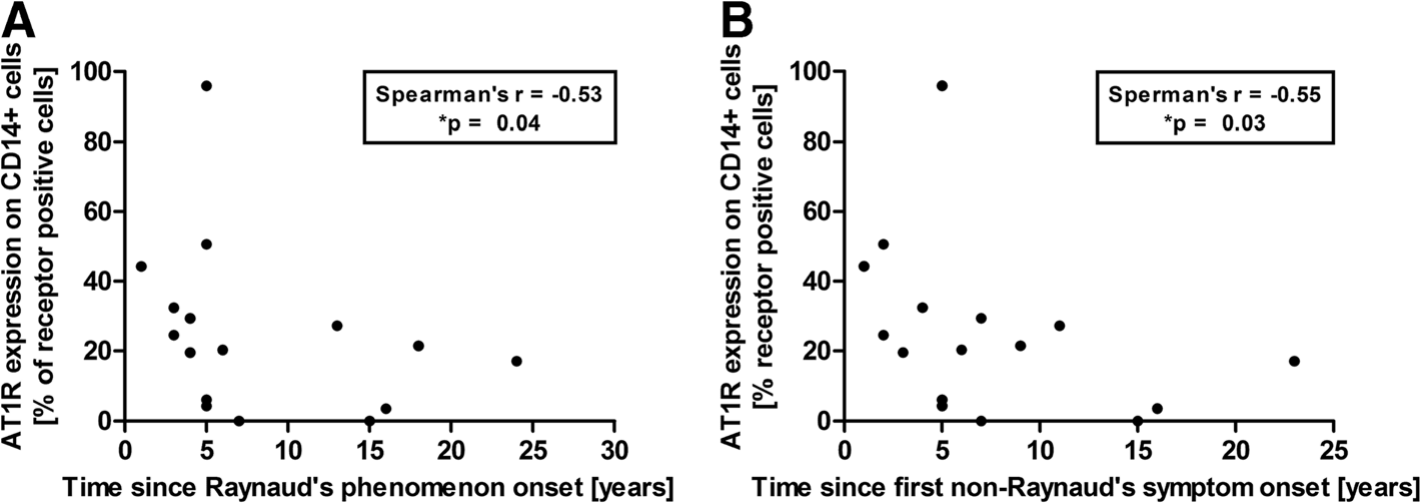
**Angiotensin II receptor type 1 protein expression in monocytes of systemic sclerosis patients correlates negatively with disease duration.** Frequency of angiotensin II receptor type 1 (AT1R)-positive CD14+ monocytes shows a significant negative correlation with **(A)** the time since onset of Raynaud’s phenomenon (patients with reported onset of Raynaud’s phenomenon; *n* = 16) and **(B)** the time since onset of the first non-Raynaud’s symptom presenting the onset of the disease (patients with reported onset of the first non-Raynaud’s symptom; *n* = 16). Statistical analysis was done by Spearman’s correlation. *P < 0.05.

In SSc patients, receptor expression on PBMCs may reflect patterns of the disease. When we compared receptor expression with clinical data, we observed negative correlations between the frequency of AT1R-positive CD14+ monocytes and the time since onset of Raynaud’s phenomenon and first non-Raynaud’s symptom (Figures 2A and 2B). Correlation analysis between the AT1R density on CD14+ monocytes and the time from the onset of Raynaud’s phenomenon and the first non-Raynaud’s symptom revealed similar results (see Additional file 1).

No further associations were found between the receptor expression data and clinical features such as disease activity, lung function parameters or the presence of further manifestations in SSc patients.

Statistical analysis of data from healthy individuals revealed a negative correlation between protein expression of both receptors and age, with statistical significance for AT1R density on CD14+ monocytes and the frequency of AT1R-positive CD14+ monocytes, and the frequency of AT1R- and ETAR-positive CD19+ B cells. In patients, no age-related association was observed (see Additional file 2).

Interestingly, in female healthy donors, both receptors were expressed at a lower level in all analyzed PBMC populations compared to those in male healthy donors (see Additional file 3). Neither in patients nor in healthy individuals AT1R expression results showed correlations with those for the ETAR or with the respective Aab levels.

### SSc-IgG-induced chemotaxis of T cells was reduced by AT1R and ETAR antagonists and correlates positively with Aab levels

As demonstrated above, PBMCs express AT1R and ETAR. Therefore, activation of PBMCs through the AT1R and the ETAR by agonistic Aabs must be supposed. To address the hypothesis that lower frequencies of AT1R- and ETAR-positive monocytes and T cells could result from increased migration into the tissue due to Aab activation in SSc patients, we performed chemotaxis assays. Initial migration experiments with freshly isolated PBMCs through a collagen-coated membrane resulted in a significantly higher number of PBMCs that migrated toward IgG of SSc patients (SSc-IgG) compared with IgG of healthy donors (HD-IgG), but the differences from the medium control were low. Migration assays of isolated monocytes provided no results, owing to the obviously high activation status of the cells. Therefore, chemotaxis assays were performed with isolated T cells. T cells migrated toward the natural ligands Ang II and ET-1 and toward SSc-IgG in a concentration-dependent manner as studied in initial experiments. Migration was dose-dependently inhibited by the AT1R antagonist valsartan and the ETAR antagonist sitaxsentan (see Additional file 4).

**Figure 3:**
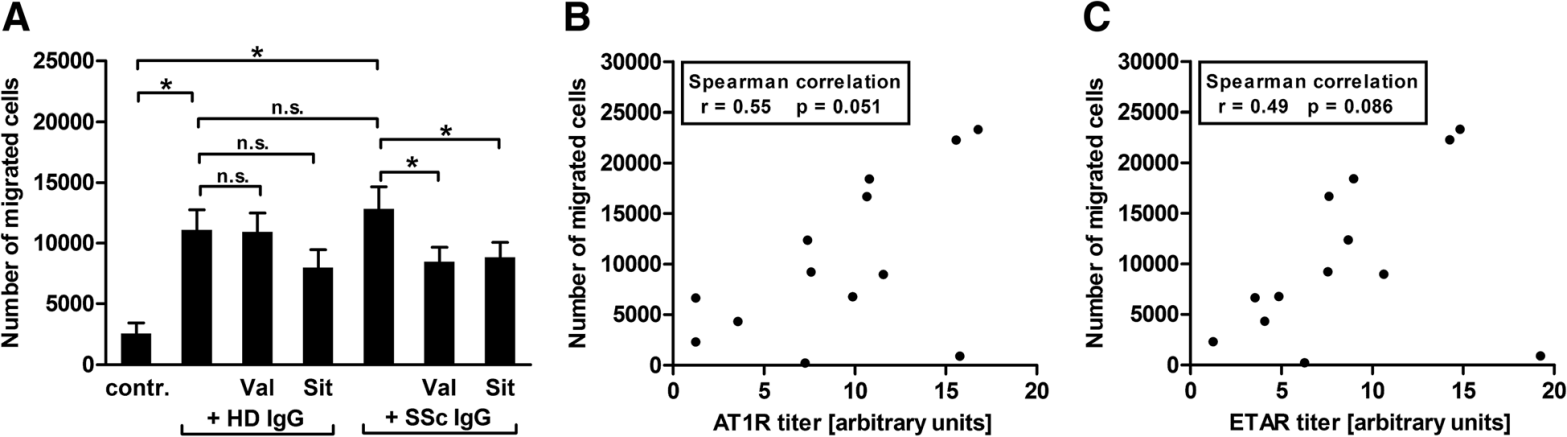
**Chemotaxis of T cells induced by immunoglobulin G from systemic sclerosis patients is reduced by receptor antagonists and depends on autoantibody levels. (A)** Number of T cells that migrated toward medium control (contr.), immunoglobulin G of healthy donors (HD-IgG) and systemic sclerosis patients (SSc-IgG). Chemotaxis induced by SSc-IgG was significantly reduced by the angiotensin II receptor type 1 (AT1R) antagonist valsartan (Val) and the endothelin receptor type A (ETAR) antagonist sitaxsentan (Sit). Chemotaxis induced by HD-IgG was not significantly reduced (n.s.). **(B)** Numbers of T cells that migrated toward SSc-IgG correlate with anti-AT1R Aab levels of the SSc-IgG fractions used. **(C)** Numbers of T cells that migrated toward SSc-IgG correlate with anti-ETAR Aab levels of the SSc-IgG fractions used. Data are derived from five independent migration assays done with a total of 13 SSc-IgG and 10 HD-IgG. Statistical analysis was done by Mann–Whitney *U* test (control vs. HD-IgG, control vs. SSc-IgG and HD-IgG vs. SSc-IgG), Wilcoxon matched-pairs test (IgG vs. IgG with antagonist) and Spearman's correlation (B and C). Mean +/- SEM are shown in (A). *P < 0.05.

As shown in Figure 3A, significantly more T cells migrated toward SSc-IgG and HD-IgG than toward the medium control. The numbers of T cells that migrated toward SSc-IgG was higher compared with HD-IgG, but this difference did not reach statistical significance. Chemotaxis induced by SSc-IgG was significantly reduced by the AT1R antagonist valsartan and the ETAR antagonist sitaxsentan, but not the chemotaxis induced by HD-IgG.

Strong correlations were found between the number of T cells that migrated toward SSc-IgG and the anti-AT1R and anti-ETAR Aab levels of the SSc-IgG fractions (Figures 3B and 3C). In contrast, the number of T cells that migrated toward HD-IgG did not correlate with Aab levels (see Additional file 5).

No further associations were found between numbers of migrated T cells and age, sex, disease form or other clinical features of IgG donors.

### PBMCs showed altered expression of cell surface markers and secretion of cytokines when incubated with IgG from SSc patients

IgG from SSc patients may induce inflammatory and fibrotic conditions. Therefore, the differential expression of activation markers, cytokines and soluble proteins was analyzed after PBMCs of healthy donors were incubated with either SSc IgG or HD IgG.

**Figure 4:**
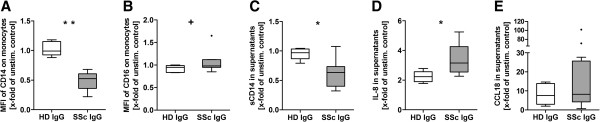
**Immunoglobulin G of systemic sclerosis patients changed expression of markers on peripheral blood mononuclear cells and secretion of cytokines.** Peripheral blood mononuclear cells (PBMCs) from healthy donors (HD) were stimulated for 8 to 20 hours *in vitro* by either immunoglobulin (IgG) from healthy donors (HD-IgG) or systemic sclerosis (SSc) patients (SSc-IgG). Protein expression of cell surface markers **(A)** CD14 and **(B)** CD16 were measured on monocytes by flow cytometry, density is represented by the median fluorescence intensity (MFI). **(C)** The soluble protein CD14 (sCD14), as well as the cytokines **(D)** interleukin 8 (IL-8) and **(E)** chemokine (C-C motif) ligand 18 (CCL18), were measured in the supernatants by enzyme-linked immunosorbent assay. Data derived from three independent experiments done with a total of five HD-IgG and ten SSc-IgG normalized to the unstimulated control. Statistical analysis was done by Mann–Whitney *U* test. Data are shown as box-and-whisker plots (Tukey). ^+^*P* < 0.1, *P < 0.05 and **P < 0.01.

In monocytes, expression of CD14 on forward scatter/side scatter–gated monocytes was significantly decreased in SSc-IgG-stimulated samples (Figure 4A), whereas CD16 on CD14+ monocytes, but not on monocytes with diminished CD14 expression (CD14^dim^), was slightly increased (Figure 4B). sCD14 in supernatant was significantly decreased, too (Figure 4C). Expression of human leukocyte antigen major histocompatibility class II cell surface receptor DR was not altered (see Additional file 6).

Incubation of PBMCs with SSc-IgG resulted in significantly increased IL-8 concentrations in supernatants when compared with incubation of PBMCs with HD-IgG (Figure 4D). In addition, CCL18 was increased in supernatants, although not significantly, but with a remarkable range up to more than 100-fold that of the control (Figure 4E), reflecting the heterogeneity of responses induced by IgG from different SSc patients. The SSc-IgG-induced TGF-β expression was only slightly increased, and sCD62 was slightly decreased, compared with the HD-IgG (see Additional file 6). The natural ligands Ang II and ET-1 did not modify the expression or secretion of the tested proteins when applied alone.

### SSc-IgG-induced IL-8 and CCL18 secretion was reduced by AT1R and ETAR antagonists

**Figure 5:**
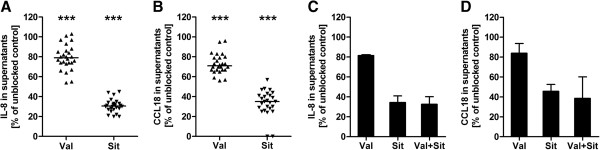
**Receptor antagonists reduced interleukin 8 and chemokine (C-C motif) ligand 18 secretion, inhibiting effects are not additive. (A)** Interleukin 8 (IL-8) and **(B)** chemokine (C-C motif) ligand 18 (CCL18) concentrations in the supernatants of peripheral blood mononuclear cells (PBMCs) from healthy donors after 8 hours of *in vitro* stimulation by immunoglobulin G of systemic sclerosis patients (SSc-IgG) after pretreatment with either the angiotensin II receptor type 1 blocker valsartan (Val) or the endothelin receptor type A blocker sitaxsentan (Sit), measured by enzyme-linked immunosorbent assay (ELISA) and normalized to the corresponding sample without any antagonist representing 100%. Data are derived from one experiment done with 26 SSc-IgG. Simultaneous blocking with both antagonists did not improve the inhibiting effects: **(C)** IL-8 and **(D)** CCL18 concentrations in the supernatants of PBMCs from three healthy donors after 8 hours of *in vitro* stimulation by SSc-IgG after pretreatment with valsartan (Val) or sitaxsentan (Sit) or both (Val + Sit) measured by ELISA and normalized to the corresponding samples without any antagonist representing 100%. Data derived from three independent experiments done with pooled IgG from thirteen SSc patients. Statistical analysis was done by Wilcoxon matched-pairs test. Medians (A and B) and medians with range (C and D) are shown. ***P < 0.001.

IL-8 and CCL18 are strong mediators of inflammation and fibrosis, respectively. Utilizing specific receptor antagonists, we analyzed whether the observed IL-8 and CCL18 responses of PBMCs induced by SSc-IgGs (Figures 4D and 4E) were mediated through AT1R and/or ETAR. Because of the high interassay variability due to different cell donors that we had observed in the migration assays, we tested at first the IgG fractions of 26 SSc patients with the cells of one donor. IL-8 secretion was decreased 21% by valsartan and 69.5% by sitaxsentan (Figure 5A). CCL18 secretion was decreased 29% by valsartan and 65% by sitaxsentan (Figure 5B). In a second approach, we examined combined treatment with both antagonists, but it did not improve the inhibiting effects in the tested dosages (Figures 5C and 5D).

### SSc-IgG-induced IL-8 concentrations correlate with clinical features

We detected a wide range of IL-8 and CCL18 levels after *in vitro* stimulation of PBMCs with SSc-IgG of several donors. Assuming a similar effect of the Aabs *in vivo*, the capability of SSc-IgG to induce IL-8 and CCL18 may correspond to clinical manifestations in the respective IgG donors. Therefore, analyses were performed to correlate the chemokine concentrations induced *in vitro* with clinical data.

**Figure 6:**
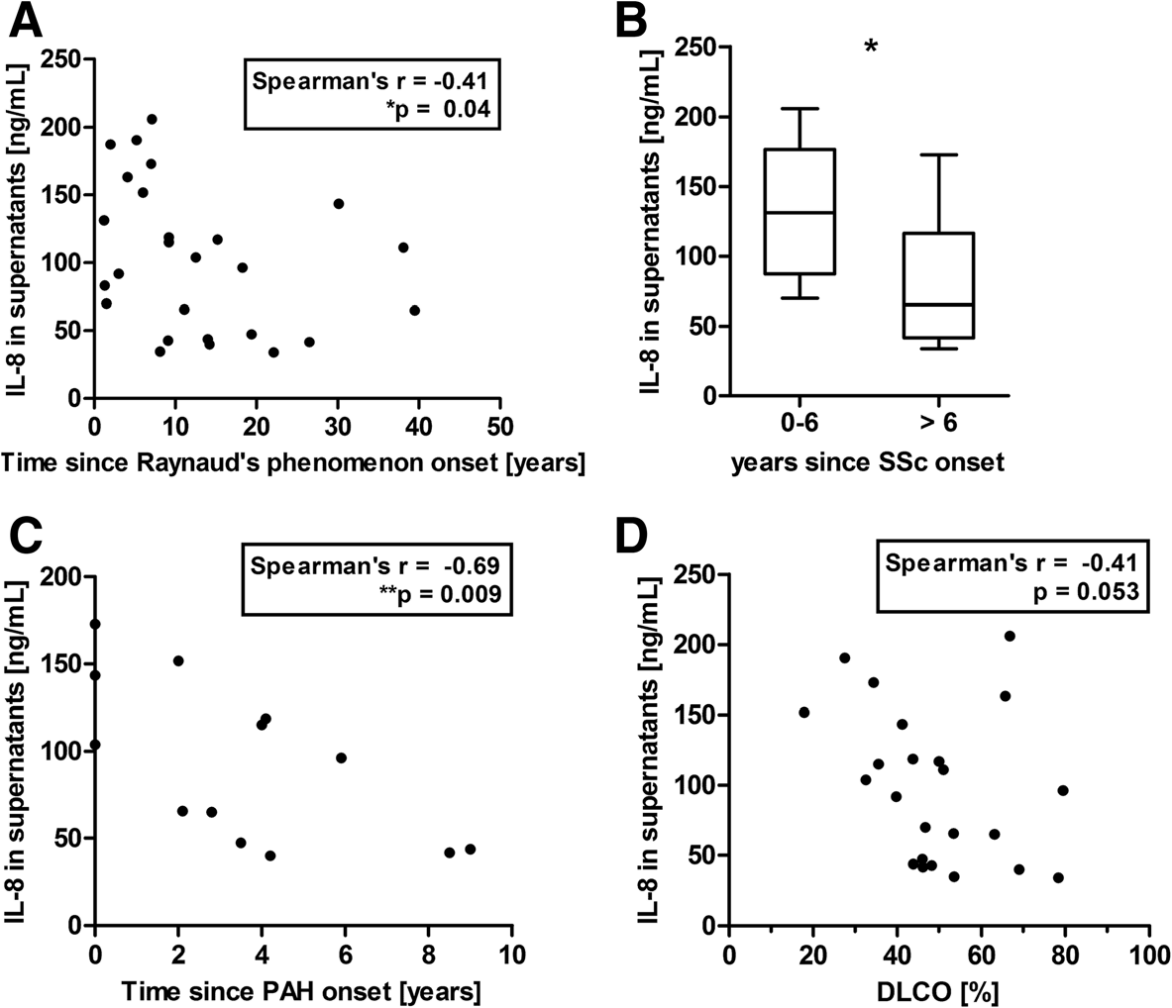
**Interleukin 8 concentrations induced by immunoglobulin G of systemic sclerosis patients (SSc-IgG) in supernatants correlate with clinical features of SSc-IgG donors. (A)** Correlation of measured interleukin 8 (IL-8) concentrations with the time since Raynaud’s phenomenon onset (26 patients with reported Raynaud’s phenomenon onset) and **(B)** classification into early and late stages of the disease (0 to 6 years and >6 years since systemic sclerosis (SSc) onset, respectively) by means of the first non-Raynaud’s symptom onset (25 patients with reported first non-Raynaud’s symptom onset). **(C)** Correlation of measured IL-8 concentrations with the time since onset of pulmonary arterial hypertension (PAH; 10 patients with reported PAH onset) and **(D)** with the diffusion capacity of the lung for carbon monoxide (DLCO; 23 patients with reported DLCO). IL-8 levels were measured by enzyme-linked immunosorbent assay. Statistical analysis was done by two-tailed Spearman's correlation (A, B and D) and by Mann–Whitney *U* test (B). Data in (B) are shown as box-and-whisker plot (Tukey). *P < 0.05, **P < 0.01.

IL-8 concentrations in supernatants of PBMCs stimulated with SSc-IgG revealed a negative correlation with the time since onset of Raynaud’s phenomenon of the respective IgG donor (Figure 6A). In line with this observation, IgG from SSc patients in an early stage of disease (0 to 6 years from the time of onset of the first non-Raynaud’s symptom) caused significantly higher IL-8 concentrations when compared with IgG levels of patients in a late stage (more than 6 years since onset) (Figure 6B). In addition, IL-8 concentrations revealed a highly significant negative correlation with the time since onset of pulmonary arterial hypertension (PAH) (Figure 6C). A strong negative correlation was observed between IL-8 concentrations in the supernatants and the diffusion capacity of the lung for carbon monoxide (DLCO) measured in lung function tests (Figure 6D). No associations were observed between IL-8 concentrations and age, sex, disease form, disease activity or other clinical features, or the presence of Aabs against centromere and Scl-70. Anti-AT1R and anti-ETAR Aab levels of patients and of affinity-purified IgG fractions did not correlate with IL-8 concentrations in supernatants or with blocking effects of valsartan and sitaxsentan. Concentrations of IL-8 and CCL18 in supernatants did not correlate with each other (see Additional file 7).

### SSc-IgG-induced CCL18 concentrations correlate with clinical features

**Figure 7:**
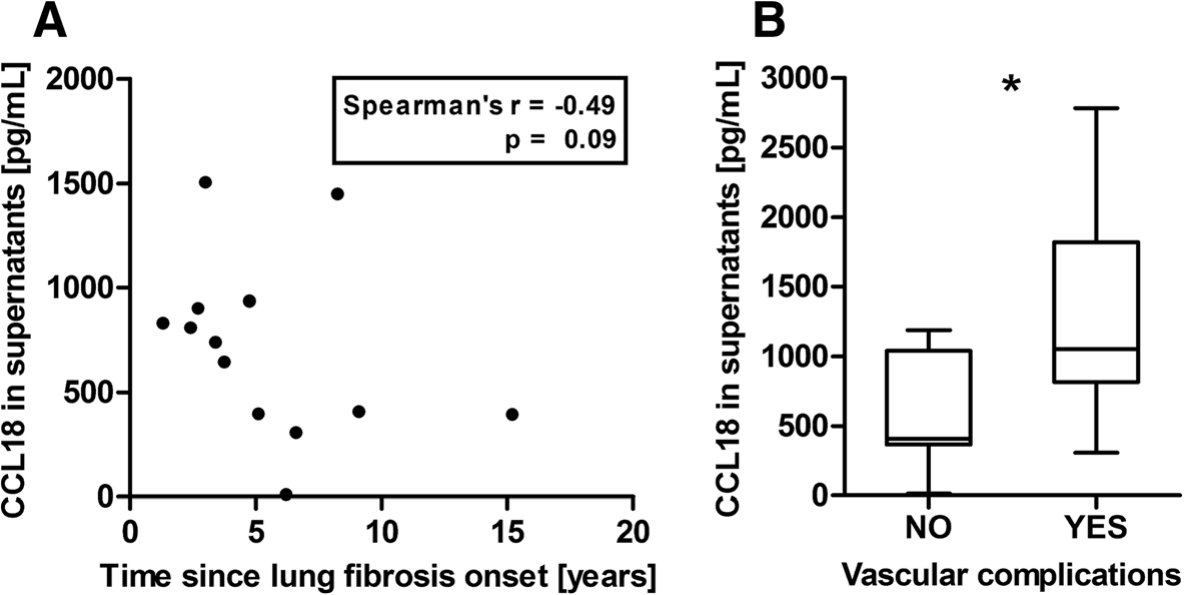
**Chemokine (C-C motif) ligand 18 concentrations induced by immunoglobulin G of systemic sclerosis patients (SSc-IgG) in supernatants correlate with clinical features of SSc-IgG donors. (A)** Correlation of measured chemokine (C-C motif) ligand 18 (CCL18) concentrations with the time since lung fibrosis onset (13 patients with reported lung fibrosis onset). **(B)** Association of measured CCL18 concentrations in supernatants with the incidence of vascular complications (25 patients with reported vascular status). CCL18 concentrations were measured by enzyme-linked immunosorbent assay. Statistical analyses were done by two-tailed Spearman's correlation (A) and Mann–Whitney *U* test (B). Data in (B) are shown as box-and-whisker plot (Tukey’s test). *P < 0.05.

Correlation analysis of CCL18 concentrations in supernatants with clinical features of SSc-IgG donors revealed a negative correlation with the time since lung fibrosis onset (Figure 7A). In addition, IgGs of SSc patients with vascular complications, including digital ulcers, PAH and renal crisis, caused significantly higher CCL18 secretion than those of patients without these complications (Figure 7B). No correlations were identified between CCL18 concentrations and time since onset of Raynaud’s phenomenon or onset of SSc, and no associations were found between CCL18 concentrations and age, sex, disease activity or other clinical features and Aab titers.

## Discussion

Expression of AT1R and ETAR has been found in several cell types, both receptors mediate the activation of various pathways, depending on the function of the relevant tissue [7-9,13-15,22]. In this study, we investigated the expression of the AT1R and the ETAR on human PBMCs of healthy individuals and SSc patients, the functional consequences of *in vitro* activation of these receptors on immune cells by agonistic Aabs and the association of the observed effects with important disease features.

### AT1R and ETAR are expressed in PBMCs and downregulated in SSc patients

In previous studies the expression of the AT1R on circulating leukocytes has been described [8,16], but not the expression of ETAR. As we have shown in the present study, the ETAR is expressed on human T cells, B cells and monocytes (Figure 1). We studied protein expression of both receptors *ex vivo* by intracellular staining, thereby avoiding alterations caused by steric impairment, internalization and conformational changes of the receptors induced by ligands or Aabs. Recently, nonspecific binding of the anti-AT1R antibody sc-1173 has been described [23]. However, we have confirmed the specificity of the antibody by the anti-AT1R antibody ELISA we performed to analyze AT1R Aabs in serum or purified IgG (see Additional file 8). In addition, expression of both receptors was confirmed on mRNA level by real-time PCR, verifying the protein expression data, where we also found the highest AT1R and ETAR expression on monocytes and T cells.

Protein expression of both receptors was decreased on PBMCs from SSc patients, especially on T cells and monocytes (Figure 1). It is well-established that Ang II and ET-1, as well as several growth factors, downregulate the expression of the AT1R and the ETAR, and that all of them are increased in the serum of SSc patients [6,24-28]. Internalization, degradation, desensitization and tolerance to agonists are classical feedback mechanisms to respond to short-term and long-term receptor activation [29,30]. We suggest that, besides natural ligands and growth factors, receptor-activating Aabs also diminish the expression of the AT1R and the ETAR in PBMCs of SSc patients in the long term. This notion is supported by our finding that receptor expression on PBMCs of SSc patients appears to decrease during the course of the disease (Figure 2). In line with this, reduced AT1R expression has previously been observed in vessels from granulomatous lesions of patients with granulomatosis due to polyangiitis (formerly known as Wegener’s granulomatosis), another vascular disease characterized by inflammation, endothelial dysfunction and Aabs that induce tissue damage [31]. Furthermore, decreased ETAR expression has been found in fibrotic lung tissue and skin-derived fibroblasts from SSc patients [24,28].

Other factors that can modulate receptor expression are cytokines, hypoxia, nitric oxide, free radicals, estrogens and progesterone [6,24-28]. Therefore, it is not surprising that we found higher expression of AT1R and ETAR in male donors than in female donors. Whether this could explain the higher susceptibility of male SSc patients to renal crisis or cardiomyopathy remains to be elucidated.

### Role of AT1R- and ETAR- activating Aabs in SSc

In the present study we observed that SSc-IgG positive for anti-AT1R and anti-ETAR Aabs induced T-cell migration as well as IL-8 and CCL18 secretion from PBMCs *in vitro*. These effects were significantly reduced by receptor antagonists. The experimental results correlate with the clinical data.

The lower frequency of the AT1R and the ETAR on PBMCs from SSc patients than that of healthy donors may be due to increased migration of receptor-positive cells. In this scenario, high levels of Ang II, ET-1 or Aabs may trigger the invasion of immune cells into the surrounding tissue. It has previously been reported that Ang II is a potent chemoattractant for different immune cells [8,18,32] and that ET-1 participates in mononuclear cell infiltration by enhancing the expression of adhesion molecules on fibroblasts [33]. In our present study, PBMCs and isolated T cells migrated toward IgG in a concentration-dependent manner (Additional file 4), with a greater number of cells migrating toward SSc-IgG than toward HD-IgG (Figure 3). The number of migrated T cells correlated with the anti-AT1R and anti-ETAR levels of the SSc-IgG fractions. In addition, T-cell migration induced by SSc-IgG, but not that induced by HD-IgG, was significantly reduced by AT1R and ETAR blockers, confirming that the migration was mediated mainly through the AT1R and the ETAR (Figure 3).

Functional antibodies against AT1R and ETAR have been described in SSc as well as in other diseases [2,34-45]. Both Aabs are biologically active and induced direct receptor-mediated, extracellular signal–regulated kinase 1/2 phosphorylation and increased profibrotic TGF-β gene expression in the human microvascular endothelial cell line 1 (HMEC-1), effects that could be diminished by the respective receptor antagonists. In addition, the Aabs were predictive of mortality and showed strong associations with fibrotic and vascular complications [2].

Thus, we hypothesized that these Aabs contribute to inflammatory and fibrotic conditions in SSc by activating the receptors on immune cells similarly to Ang II and ET-1. Ang II is involved in all stages of vascular inflammation [9] by inducing several transcription factors and releasing different cytokines and chemokines, such as IL-8 [34,46-50]. In addition, Ang II is involved in fibrosis, whereby the AT1R acts as a profibrotic mediator and the AT2R acts as an antifibrotic mediator [10]. Among the transcription factors induced by Ang II, nuclear factor κB (NF-κB) plays a crucial role in regulating several proinflammatory genes, including the IL-8 gene [47]. Ang II–induced inflammation via NF-κB seems to involve ET-1 as well. ET-1 is a powerful mediator of inflammation in the vasculature [13], and the addition of an ET-1 receptor antagonist decreased NF-κB and inflammation in Ang II–induced end-organ damage [51]. Thus, Ang II–induced vascular inflammation might be amplified by cross talk with the endothelin pathway.

In the present work, we used SSc-IgG fractions positive for anti-AT1R and anti-ETAR Aabs to analyze their pathological effects on human monocytes and lymphocytes *in vitro*, because the innate and the adaptive immune systems both have a role in the pathogenesis of SSc [4]. We found a significantly decreased CD14 expression on monocytes after incubation with SSc-IgG that was not a result of a shedding effect, as shown by sCD14 ELISA results (Figure 3). In addition, there was slightly increased CD16 expression, but not on the CD14^dim^ population. Therefore, we could not ascribe these surface marker alterations to an activation or a known differentiation type. Moreover, a desensitizing effect may have occurred. This interesting effect needs to be studied in more detail in the future.

TGF-β is a main profibrotic factor, produced by lymphocytes and monocytes among the PBMCs. On the basis of our experience with endothelial cells (HMEC-1), we expected SSc-IgG to trigger a strong TGF-β response [2]. Instead, we found that SSc-IgG-stimulated PBMCs secreted high levels of IL-8 and CCL18. IL-8 is a major inflammatory cytokine produced mainly by endothelial cells and monocytes/macrophages. It acts as a chemoattractant to neutrophils, T cells and monocytes. Thus, Aabs evoking a strong IL-8 response can contribute to the perivascular leukocyte infiltration, such as that observed in the early stages of SSc [6,52]. In addition, IL-8 is able to activate fibroblasts, thereby promoting fibrosis [53]. CCL18 is a prototypic fibrotic cytokine released mainly by monocytes/macrophages and dendritic cells. It acts as chemoattractant to lymphocytes and is constitutively present in human plasma, contributing to the generation of primary immune responses [54]. Enhanced CCL18 production has been verified in several inflammatory skin and lung diseases [55]. Monocytes of SSc patients and patients with pulmonary fibrosis are known to express more CCL18 than those of healthy donors [56,57]. Both CCL18 and Ang II are able to induce fibrotic effects via TGF-β-independent signaling pathways [54,58-60]. Thus, Aab-mediated CCL18 release can contribute to profibrotic conditions independently of TGF-β.

Interestingly, although Ang II is able to induce the release of different cytokines such as IL-8 in other cell types [46-50], no stimulatory effect was found without costimulation when the effect of Ang II on T cells, has been studied [7,17]. In line with this observation, and unlike Aabs, we were unable to induce IL-8 and CCL18 expression in PBMCs by Ang II and ET-1 alone. Taken together, the importance of costimuli for T-cell receptor activation and cross talk between AT1R and ETAR could explain the different effects of Aabs and natural ligands.

By pretreating cells with the pharmacological receptor antagonists valsartan and sitaxsentan, we found a significant reduction of SSc-IgG-induced IL-8 and CCL18 concentrations in the supernatants of PBMCs. This finding supports our hypothesis that anti-AT1R and anti-ETAR Aabs mediate inflammatory and profibrotic effects via an activation of their respective receptor. Both antagonists were able to inhibit IL-8 and CCL18 release, suggesting a direct association between either the release of the cytokines or the effects of the two different Aabs. Correlation analysis between IL-8 concentrations and the respective CCL18 concentrations revealed a low negative regression coefficient, implying that CCL18 does not account for IL-8 release or vice versa. Moreover, IgGs from SSc patients induced high levels of either CCL18 or IL-8. Inhibition of the AT1R by its respective antagonist, however, reduced IL-8 and CCL18 secretion to a similar extent. Also, the inhibition of the ETAR by its respective antagonist reduced IL-8 and CCL18 secretion similarly. The underlying mechanism of these observations is highly speculative. One idea might be that the downstream signaling events of both receptors have a crossing point which confers the signal for IL-8 and CCL18 production. Inhibition of the AT1R was less efficient than the inhibition of ETAR. This might be a result of varying affinities and avidities of the AT1R- and ETAR-activating Aabs, respectively.

### Aab-induced IL-8 and CCL18 concentrations correlate with clinical findings of SSc-IgG donors

In our study, we analyzed the IL-8 and CCL18 secretion of PBMCs after *in vitro* stimulation with SSc-IgGs positive for anti-AT1R and anti-ETAR Aabs. Assuming that these antibodies also play a pathological role *in vivo*, Aab-induced IL-8 and CCL18 concentrations were correlated to clinical findings in the donor patients. First, there was a significant negative correlation between IL-8 concentrations with the time since Raynaud’s phenomenon onset and PAH onset, suggesting that the inflammatory activity of the Aabs is high at the onset of certain disease manifestations and decreases during the course of disease. This observation is in accord with the high IL-8 levels in the affected skin of patients in the early stages of SSc [61]. Changes in the avidity and/or affinity of Aabs after disease onset have been described previously [62] and remain to be studied further in the future. Second, the negative correlation of IL-8 concentrations with the DLCO seems to be independent of the course of the disease and may point out a direct association between the capacity of the Aabs to induce IL-8 secretion and the worsening of DLCO. This has been suggested by previous work showing a significant negative correlation between DLCO and IL-8 levels measured in bronchoalveolar lavage fluid from SSc patients [63]. Third, high CCL18 concentrations were associated with vascular complications, including digital ulcers, PAH and renal crisis, and correlated negatively with the time since lung fibrosis onset, suggesting that the profibrotic activity of the Aabs is higher at the beginning of lung fibrosis. All of these complications result from fibrotic alterations, where CCL18 is known to be highly involved [64,65]. Therefore, these findings support the idea of an inflammatory and profibrotic activity of anti-AT1R and anti-ETAR Aabs by IL-8 and CCL18 induction.

### Limitation

The limitation of this study is the lack of purified Aabs against AT1R and ETAR. Generation of functional monoclonal antibodies against AT1R and ETAR, as well as against other GPCRs, is a general problem unsolved to date. Owing to the high volume of serum necessary for specific autoantibody isolation, we isolated the whole IgG fractions from sera of patients with that severe disease and proved the specificity of AT1R- and ETAR-mediated effects by the application of specific receptor antagonists. Some of the Aab-induced IL-8 and CCL18 responses were not blockable, suggesting possible involvement of antibodies directed toward other targets involved in IL-8 and CCL18 pathways.

## Conclusions

In our present study, we have demonstrated that not only the previously identified AT1R but also the ETAR is expressed on human peripheral T cells, B cells and monocytes. Therefore, these immune cells are under direct control of the natural ligands Ang II and ET-1 as well as of receptor-activating Aabs. In SSc patients, we found a distinctly decreased receptor expression, probably reflecting chronic activation. AT1R- and ETAR-mediated T-cell migration and high IL-8 and CCL18 responses in immune cells due to stimulation by receptor-activating Aabs support our hypothesis that these Aabs contribute to an inflammatory and profibrotic environment in the perivascular tissue. The high inflammatory response induced by Aabs of SSc patients in the early disease stages further suggests a prominent role of Aabs in the initiation of SSc as well as a possible benefit of receptor blockade in the early stages of the disease.

## Abbreviations

Aabs: Autoantibodies; Ang II: Angiotensin II; AT1R: Angiotensin II receptor type 1; AT2R: Angiotensin II receptor type 2; BSA: Bovine serum albumin; CCL18: Chemokine (C-C motif) ligand 18; CD: Cluster of differentiation; CXCL8: Chemokine (CXC motif) ligand 8; DLCO: Diffusion capacity of the lung for carbon monoxide; ELISA: Enzyme-linked immunosorbent assay; ET-1: Endothelin 1; ETAR: Endothelin receptor type A; GPCR: G protein–coupled receptor; HD-IgG: Immunoglobulin G of healthy donors; HMEC-1: Human microvascular endothelial cell line 1; IgG: Immunoglobulin G; IL-8: Interleukin 8; MFI: Median fluorescence intensity; PAH: Pulmonary arterial hypertension; PBMC: Peripheral blood mononuclear cell; PBS: Phosphate-buffered saline; sCD14: Soluble cluster of differentiation 14; sCD62: Soluble cluster of differentiation 62; SSc: Systemic sclerosis; SSc-IgG: Immunoglobulin G of systemic sclerosis patients; TGF-β1: Transforming growth factor β1.

## Competing interests

The authors declare that they have no competing interests.

## Authors’ contributions

JG performed the experiments, data analyses and statistical analyses and wrote the manuscript. AK performed the real-time PCRs. HH participated in the migration assays and analyzed the Aab levels. JR participated in the analysis of protein expression. MB designed the study. ES helped to write the manuscript. MR, GB and DD contributed to the systematic review. GR conceived of the study aims, participated in the study design and contributed to the systematic review. All authors read and approved the final manuscript.

## Supplementary Material

Additional file 1 **Angiotensin II receptor type 1 protein expression in monocytes of systemic sclerosis patients correlates negatively with disease duration.** Density of angiotensin II receptor type 1 (AT1R) on CD14+ monocytes correlates negatively with **(A)** the time since onset of Raynaud’s phenomenon (16 patients with reported onset of Raynaud’s phenomenon) as a trend and **(B)** the time since onset of the first non-Raynaud’s symptom presenting the onset of the disease (16 patients with reported onset of the first non-Raynaud’s symptom). Statistical analysis was done by Spearman's correlation. MFI, Median fluorescence intensity. Click here for file

Additional file 2 **Expression of both receptors correlates negatively with age of healthy donors. (A)** Angiotensin II receptor type 1 (AT1R) density on CD14+ monocytes, **(B)** frequency of AT1R-positive CD14+ monocytes, **(C)** frequency of AT1R-positive CD19+ B cells and **(D)** endothelin receptor type A (ETAR)-positive CD19+ B cells in healthy donors (HD, upper panel) show a significant negative correlation with age. In systemic sclerosis patients (SSc, lower panel), no age-related association was observed. Statistical analysis was done by Spearman's correlation. *P < 0.05 and **P < 0.01. Click here for file

Additional file 3 **Angiotensin II receptor type 1 and endothelin receptor type A protein expression is lower on peripheral blood mononuclear cells of healthy women than in those of healthy men.** Protein expression of both receptors in CD3+ T cells, CD19+ B cells and CD14+ monocytes of healthy women (*n* = 8) and healthy men (*n* = 6) was measured by flow cytometry. **(A)** Density of the angiotensin II receptor type 1 (AT1R) and **(B)** density of the endothelin receptor type A (ETAR) is represented by the median fluorescence intensity normalized to the isotype control. **(C)** Frequency of AT1R-positive cells and **(D)** frequency of ETAR-positive cells is represented by the percentage relative to an isotype control. Statistical analysis was done by Mann–Whitney *U* test. MFI, Median fluorescence intensity. Data are shown as box-and-whisker plots (Tukey). **P < 0.01. Click here for file

Additional file 4 **Angiotensin II, endothelin 1 and immunoglobulin G of systemic sclerosis patients induced chemotaxis of T cells, which was reduced with receptor antagonists. (A)** Number of T cells that migrated toward medium control, angiotensin II (Ang II) and endothelin 1 (ET-1), as well as toward Ang II with different concentrations of Ang II receptor type 1 (AT1R) antagonist valsartan and toward ET-1, with different concentrations of endothelin receptor type A (ETAR) antagonist sitaxsentan. **(B)** Number of T cells that migrated toward medium control and toward immunoglobulin G of systemic sclerosis patients (SSc-IgG) at different concentrations. **(C)** Number of T cells that migrated toward medium control and toward 0.5 mg/ml SSc-IgG at different concentrations of the AT1R antagonist valsartan and the ETAR antagonist sitaxsentan. Data are summarized from four independent test experiments Mean +/- SEM are shown. Click here for file

Additional file 5 **Chemotaxis of T cells induced by immunoglobulin G of healthy donors is independent of autoantibody levels. (A)** Numbers of T cells that had migrated toward HD-IgG do not correlate with levels of autoantibodies (Aab) against angiotensin II receptor type 1 (AT1R) of the immunoglobulin G fractions of healthy donors (HD-IgG) used. **(B)** Numbers of T cells that migrated toward HD-IgG did not correlate with levels of Aab against endothelin receptor type A (ETAR) of the HD-IgG fractions used. Data derived from five independent migration assays done with a total of ten HD-IgG. Statistical analysis was done by Spearman's correlation. Click here for file

Additional file 6 **Immunoglobulin G from systemic sclerosis patients did not change the expression and/or concentration of human leukocyte antigen major histocompatibility class II cell surface receptor DR (HLA-DR), transforming growth factor β1 and soluble CD62L.** Peripheral blood mononuclear cells from healthy donors (HD) were stimulated for 8 hours *in vitro* by either immunoglobulin G from HD (HD-IgG) or IgG from systemic sclerosis patients (SSc-IgG). Protein expression of the cell-surface marker human leukocyte antigen major histocompatibility class II cell surface receptor DR (HLA-DR) **(A)** was measured by flow cytometry. Density is represented by the median fluorescence intensity (MFI). The levels of the cytokine transforming growth factor β1 (TGF-β1) **(B)** and the soluble protein CD62L (sCD62L) **(C)** were measured in the supernatants by enzyme-linked immunosorbent assay. Data are derived from three independent experiments done with a total of five HD-IgG and ten SSc-IgG, normalized to the unstimulated control. Statistical analysis was done by Mann-Whitney *U* test. Data are shown as box-and-whisker plots (Tukey). Click here for file

Additional file 7 **No correlation exists between interleukin 8 and chemokine (C-C motif) ligand 18 concentrations in supernatants after stimulation by immunoglobulin G of systemic sclerosis patients.** Interleukin 8 (IL-8) and chemokine (C-C motif) ligand 18 (CCL18) concentrations in the supernatants of peripheral blood mononuclear cells from healthy donors after 8 hours of *in vitro* stimulation by immunoglobulin G of systemic sclerosis patients (SSc-IgG) were measured by enzyme-linked immunosorbent assay. Data from one experiment with 26 SSc-IgG. Statistical analysis was done by Spearman's correlation. Click here for file

Additional file 8 **Specificity of the anti-Angiotensin II receptor type-1 antibody sc-1173 (N10) from Santa Cruz Biotechnology was tested using a commercially available sandwich enzyme-linked immunosorbent assay (One Lambda).** Plates were coated with either membrane extracts from transfected Chinese hamster ovary (CHO) cells overexpressing human angiotensin II receptor type 1 (AT1R+) or with membrane extracts from nontransfected CHO cells as controls (AT1R-). Anti-AT1R antibody sc-1173 was applied in serial dilution as indicated. OD, Optical density. Click here for file
