# Supplementary figures and images for: Angiotensin receptor type 1 and endothelin receptor type A on immune cells mediate migration and the expression of IL-8 and CCL18 when stimulated by autoantibodies from systemic sclerosis patients
Source: Arthritis Res Ther. 2014 Mar 11;16(2):R65. doi: 10.1186/ar4503 (PMC4060229; doi:10.1186/ar4503)

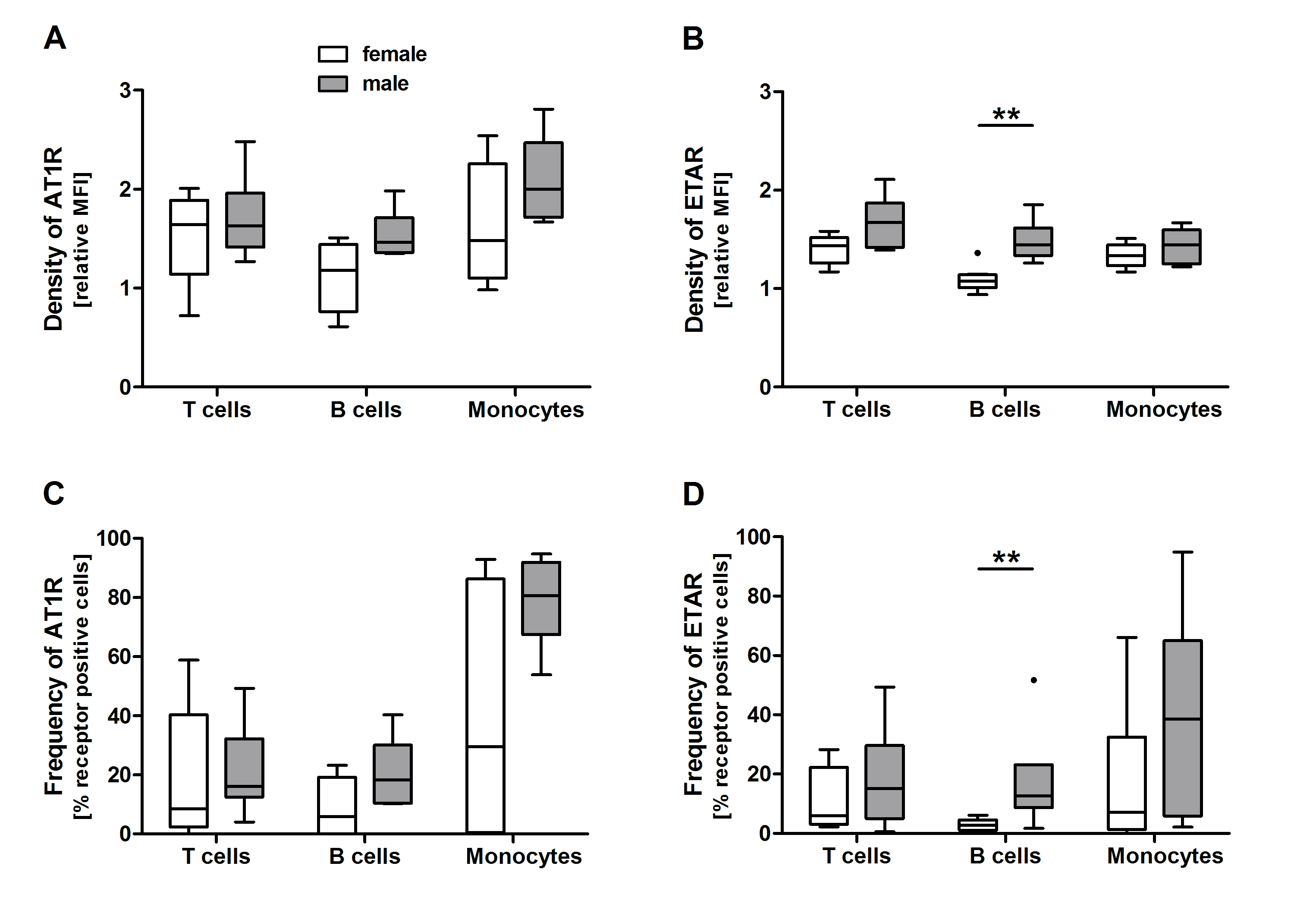

Supplement: Additional file 3 — Angiotensin II receptor type 1 and endothelin receptor type A protein expression is lower on peripheral blood mononuclear cells of healthy women than in those of healthy men. Protein expression of both receptors in CD3+ T cells, CD19+ B cells and CD14+ monocytes of healthy women (n = 8) and healthy men (n = 6) was measured by flow cytometry. (A) Density of the angiotensin II receptor type 1 (AT1R) and (B) density of the endothelin receptor type A (ETAR) is represented by the median fluorescence intensity normalized to the isotype control. (C) Frequency of AT1R-positive cells and (D) frequency of ETAR-positive cells is represented by the percentage relative to an isotype control. Statistical analysis was done by Mann–Whitney U test. MFI, Median fluorescence intensity. Data are shown as box-and-whisker plots (Tukey). **P < 0.01. [file ar4503-S3.tiff]

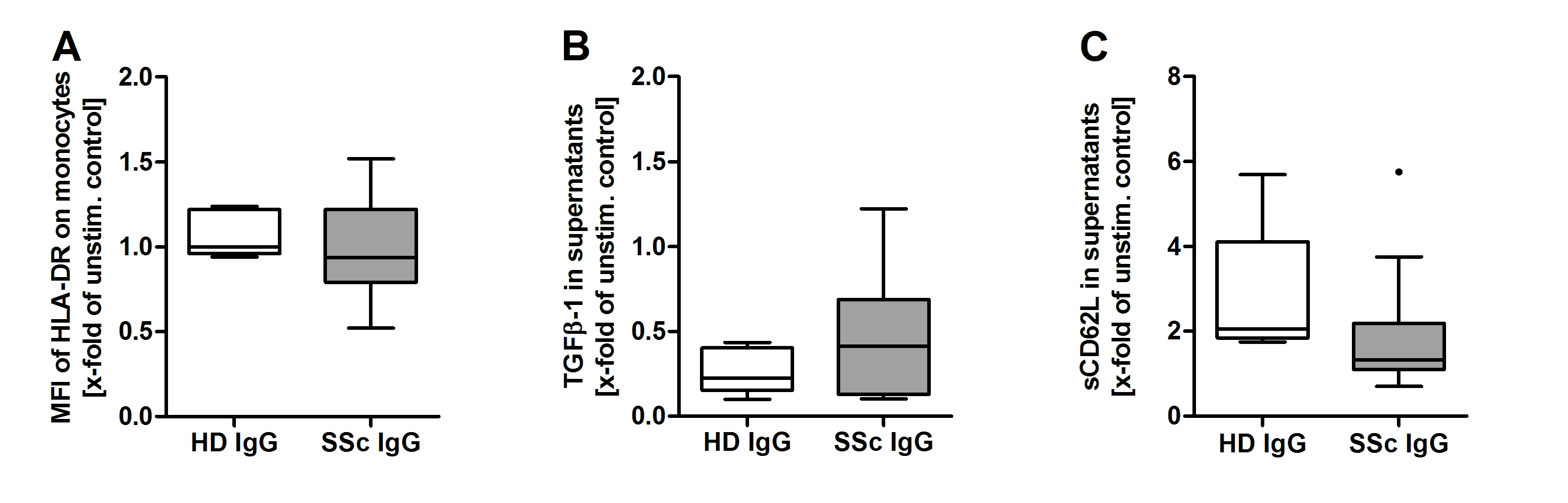

Supplement: Additional file 6 — Immunoglobulin G from systemic sclerosis patients did not change the expression and/or concentration of human leukocyte antigen major histocompatibility class II cell surface receptor DR (HLA-DR), transforming growth factor β1 and soluble CD62L. Peripheral blood mononuclear cells from healthy donors (HD) were stimulated for 8 hours in vitro by either immunoglobulin G from HD (HD-IgG) or IgG from systemic sclerosis patients (SSc-IgG). Protein expression of the cell-surface marker human leukocyte antigen major histocompatibility class II cell surface receptor DR (HLA-DR) (A) was measured by flow cytometry. Density is represented by the median fluorescence intensity (MFI). The levels of the cytokine transforming growth factor β1 (TGF-β1) (B) and the soluble protein CD62L (sCD62L) (C) were measured in the supernatants by enzyme-linked immunosorbent assay. Data are derived from three independent experiments done with a total of five HD-IgG and ten SSc-IgG, normalized to the unstimulated control. Statistical analysis was done by Mann-Whitney U test. Data are shown as box-and-whisker plots (Tukey). [file ar4503-S6.tiff]
